# Supplementary material for: The Triangulation WIthin a STudy (TWIST) framework for causal inference within pharmacogenetic research
Source: PLoS Genet. 2021 Sep 8;17(9):e1009783. doi: 10.1371/journal.pgen.1009783 (PMC8452063; doi:10.1371/journal.pgen.1009783)
Supplement: Attachment — Submitted filename: review_PGENETICS-D-21-00581.pdf [file pgen.1009783.s004.pdf]

Review of "The Triangulation WIthin A STudy (TWIST) framework for causal inference within Pharmacogenetic research"

This paper considers inference for the effect heterogeneity due to a genetic variation from data where the exposure is possibly confounded. The primary aim of the paper is to propose methods for internal replication of the inference so that multiple uncorrelated analyses are considered for the same estimand whose validity depend on different assumptions. The authors call this framework 'Triangulation Within a Study'. Although, in the literature the formalization of this framework has been called Evidence Factors analysis; see the book *Replication and Evidence Factors in Observational Studies* and the references therein.

I agree with much of the presentation and analyses of the paper, but I have several major concerns that I list below.

Major Comments:

1. **Definition of the estimand.** I am confused by how the GMTE estimate is defined in section 2, equation (2). I think the definition does not match with what the estimand is supposed to be as explained in the text.

   - What is 'g' in equation (2)? This seems like a typo and should be corrected.
   - If the estimand is defined using these marginal expectations in (2), then it would depend on the exposure distribution, i.e., the effect on the population will be attenuated when a small fraction of the population receives the treatment. Although, as I understand, this is not the aim of this estimand.

2. **Model (3).** You need to make it clear if this parametric model specification is a toy model used for illustration or an assumption for your analyses and results. From the appendices, it seems like this is an assumption for all your technical results.

   Additionally, when you move from the potential outcomes notation to the observed variable Y, please specify the appropriate SUTVA.

3. After the GMTE estimand is defined using the potential outcomes notation, under the parametric model you need to show that the estimand equates to $(\beta_1 - \beta_0)$.

   In this case, you need to be careful if assumptions are needed to make this connection.

4. **Proofs in the Appendices.**

   - In Appendices 1, 2, and 4, 'Under model (5) ...' should be 'Under model (3) ...'.

- In Appendix 1 (also 6), $\beta_{GMTE(1)}(\cdot)$ is used without definition.
- In Appendix 2, the condition $Cov(U, G) \perp\!\!\!\perp T$ is trivial and incorrect; $Cov(U, G)$ is just a number. (also in Table 1).
- In Appendix 1, $\hat{\beta}_{GMTE(0)}$ is consistent for 0 (NOT β_{GMTE}) under PG3 and $G \perp\!\!\!\perp U \mid T = 0$. (It should not be in Table 1)

5. **Homogeneity.** I would like a little more clarity regarding how the term 'Homogeneity' is used in the paper. Treatment effect homogeneity typically means the treatment effect does not change with an individual. That is, in model (3), treatment effect homogeneity means that $\beta_1 = \beta_0$.

   The paper however uses 'Homogeneity' to mean that the treatment effect is only present in the $G = 1$ group, i.e., $\beta_0 = 0$. Thus, I think clarification is needed.

6. **Combined estimators – precision vs robustness.** The combination method proposed in Section 2.5 aims to improve the precision of the combined estimate when the individual estimates are deemed similar. This is clearer in the smaller standard errors of the combined estimates relative to the individual estimates in Tables 5 and 6. Given the goal of the framework is to improve the robustness of the inference due to possible violations of variety of assumptions, I wonder if this is the correct goal.

   If we had a hypothesized value of the estimand, and robustness was the goal, we would not reject it if was not rejected by any of the individual analysis. On the other hand, we would reject a proposed value of the estimand with increased confidence if it is rejected by multiple independent analyses that depend on assumptions that do not completely overlap.

   Thus, by the duality of CI and testing it seems a robust CI of the estimand by combining multiple estimates should be wider, not narrower.

   Perhaps a concise discussion is needed to clarify the goal of the combination strategy proposed in the paper.

7. **Simulation.** In table 3 please report for each combination how many times they were deemed to be similar so they can be combined using your assessment by the Q statistic.

Minor Comments:

1. Extra 'the' in the first sentence of the second paragraph on page 2.

2. In the same paragraph, fill in the missing **REF**.

3. In equation (6), use $\hat{\beta}_{GMTE(0)}$ for $\beta_{GMTE(0)}$.

4. On page 14, 'The e_4e_4 group is now ...' to 'The e4e4 group is now ...'. Please be consistent elsewhere, e.g. §4.2.

5. In the last but one paragraph of the Discussion, ‘...both analyses after collating a much larger ...’ to ‘...both analyses after collecting a much larger ...’.

6. It might be good to acknowledge some of the literature on Evidence factors.
