# Supplementary material for: The Triangulation WIthin a STudy (TWIST) framework for causal inference within pharmacogenetic research
Source: PLoS Genet. 2021 Sep 8;17(9):e1009783. doi: 10.1371/journal.pgen.1009783 (PMC8452063; doi:10.1371/journal.pgen.1009783)
Supplement: Attachment — Submitted filename: review_PGENETICS-D-21-00581_R1_reviewer.pdf [file pgen.1009783.s006.pdf]

Review of "The Triangulation WIthin A STudy (TWIST) framework for causal inference within Pharmacogenetic research"

The authors have done a very good job in revising the paper incorporating the suggestions from the AE and the four reviewers. The revised methods section is more accessible yet technically sound. Overall, the paper highlights the importance of reporting multiple independent perfective to an effect estimation in an observational study when possible. I hope more researchers will buy into the usefulness of similar types of analyses.

I have the following minor comments.

1. On page 1, 'if the purpose of the analysis is **is** instead ...'
2. On the last paragraph of page 6, 'The Hom assumption is violated when β_0' is incomplete.
3. Third paragraph of page 7, 'In Scenario 2 the NUC ... but all others (PG1, PG2, Hom) are satisfied.' Add PG3 to the second list?
4. Section 4.3 should be part of Section 4.2, not a separate section.
5. In section 4.3.1, regarding the sentence "Its magnitude is so large ... that we can infer ... is again not sufficient ...", I do not think we can make the definitive statement that NUC is violated. I would reword this sentence.
6. In the two data analyses in Section 4, multiple p-values are reported for each study without any adjustments. Although there is a remark regarding FWER control in the Discussion section, I think it is good to add a remark on multiplicity in section 4 itself.

   Since some of these estimates are uncorrelated with each other, multiplicity adjustment is arguably more needed here when they are reported individually one after another.
