# Supplementary material for: The Triangulation WIthin a STudy (TWIST) framework for causal inference within pharmacogenetic research
Source: PLoS Genet. 2021 Sep 8;17(9):e1009783. doi: 10.1371/journal.pgen.1009783 (PMC8452063; doi:10.1371/journal.pgen.1009783)
Supplement: Attachment — Submitted filename: PGENETICS-D-21-00581_reviewer.docx [file pgen.1009783.s003.docx]

Summary:

The paper reviews the limitations that underlie pharmacogenetic approaches that rely on only treated individuals to estimate “genetically mediated treatment effect” (GMTE). The authors propose that when certain assumptions are violated, the RGMTE estimate that leverages information from untreated individuals can be used to correct for certain biases. The authors propose a framework where uncorrelated estimates, which are derived from methods with different underlying assumptions, that are observed to be similar could be combined to improve efficiency. Through a simulation study, the authors provide empirical evidence that verifies the assumptions behind each strategy, and show that their proposed RGMTE estimate is the most robust. Finally, the authors illustrate their approach using two examples: CYP2C19 genetic variants that mediate Clopidogrel efficacy and APOE genetic variants that mediate statin efficacy. This work clearly presents the limitations/assumptions of each GMTE estimation method and provides a practical decision framework for pharmacogenetic researchers to decide on the best strategy. However, further clarifications are needed to address concerns related to simulation results and potential assumption violations in the APOE-statins example. The authors should also expand on examples of prior knowledge that may suggest the violation of assumptions.

Comments:

1. Under scenario 2 of the simulation (NUC violation) presented in Table 2, the MR estimate is unbiased but has a very large standard error. Please explain the reason behind this, and clarify whether this has any implications on the use of the MR estimate or the combined RGMTE/MR estimate under such a scenario?
2. Under scenario 4 of the simulation (PG1 and NUC violation) presented in Table 2, the MR estimate is unbiased while the RGMTE estimate is biased. However, Table 3 shows that the combined RGMTE/MR estimate is unbiased (highlighted in bold). Please explain the reason behind this, and clarify whether the approach to combine estimates is still valid under the scenario where one of the estimates is biased due to the violation of assumption(s)?
3. In section 4.1, CYP2C19 LoF carriers were compared to non-carriers with regards to the risk of ischemic stroke whilst on Clopidogrel. Please clarify if non-carriers include CYP2C19 gain-of-function (e.g. CYP2C19*17) carriers? If so, the estimates would be more interpretable if gain-of-function carriers are excluded.
4. Table 6 shows that the frequency of the e2e3 and e3e4 APOE genotype is different in statin users and non-statin users. Please explain if this has any implications on the validity of the different approaches and whether this suggests a violation of the PC1 assumption?
5. In the discussion section, the authors mentioned that “expert knowledge and prior evidence should also be leveraged to decide whether the particular assumptions of the causal estimation strategy are likely to be met”. It would be very helpful to expand on this by providing examples. For example, if a variant is known to be associated with the outcome, then the PG3 assumption required for CAT and MR is likely violated, and the RGMTE estimate should be favored. Also, if it is known that G=0 individuals still benefit from treatment, then the homogeneity assumption is likely violated, and one needs to pay attention to the PG1 assumption when using the MR estimate. Relating to comment 4, could one then check if the PG1 assumption may be violated?

Minor Comments

1. The simulation results suggest that for RGMTE to be unbiased when either NUC or PG1 are met. For consistency and readability, it would be helpful if this is explicitly mentioned in Table 1 and the top left panel of Figure 2.
2. There is most likely an error on the bottom right panel of Figure 2. *Q* > χ2 *_GMTE_*_(1)_*_,CAT_* _1,1− α_ . should be replaced with *Q* < χ2 *_GMTE_*_(1)_*_,CAT_* _1,1− α_ .
3. There is a typo in Section 4.3 (Page 13): “For brevity, we focus on the results of the e2e2 versus e3e3…” e2e2 should be replaced with e2e3.
