# Supplementary material for: The Triangulation WIthin a STudy (TWIST) framework for causal inference within pharmacogenetic research
Source: PLoS Genet. 2021 Sep 8;17(9):e1009783. doi: 10.1371/journal.pgen.1009783 (PMC8452063; doi:10.1371/journal.pgen.1009783)
Supplement: Attachment — Submitted filename: Response to reviewers.docx [file pgen.1009783.s005.docx]

Response to reviewers

Dear Editor

We thank you and the reviewers for assessing our manuscript. The comments we received were excellent. They have enabled us to improve the presentation of our manuscript in this revised version and highlight important limitations in our approach to address in future research.

Please find a point-by-point response to the reviewers’ comments. Changes to the manuscript have been highlighted in blue for convenience. The main changes are

- A completely re-written methods section in which key assumptions are introduced and discussed, with most equations moved to Supporting Information 1 and Table 1. We hope that this satisfies the needs of the associate editor who asked for more accessible language and the reviewers who asked for more technical detail;
- A streamlined selection of Figures with fewer (but hopefully clearer) causal diagrams;
- Additional simulation studies to assess the ability of the heterogeneity test statistic to identify similar estimates and to assess sensitivity of estimates to decreasing allele frequency
- An extensive limitations section to our discussion.
- Many minor corrections to the wording an equations

Yours sincerely

Professor Jack Bowden (on behalf of the author team)

**Editor comments:**

*The reviewers agree that within-study triangulation is an important part of the literature and the authors' contribution is useful. The presented decision framework is novel and a key first step, which should be further developed in the future (to be able to associate rigorous type I error rate to decisions based on such trees). Reviewers also appreciated the relevance of the application.*

- Thank you for the positive assessment of our manuscript

*However, all reviewers raised important points to be addressed and several key clarifications to be able to better judge the advances this work represents. Since the target audience of the paper may be quite narrow, in order to reach out to the wide readership of PLoS Genetics, the languages could be made more accessible.*

- Thank you for this suggestion, we have tried to make the paper’s language more accessible and less technical, taking out many of the equations in the methods section from the text and inserting them into the technical appendix for the interested reader. Some of the reviewers’ comments ask for a more (rather than less) technical description of the approach. We have tried to address these whilst bearing your suggestion in mind.

**Comments to the Authors:**

**Reviewer #1:**

*This paper considers inference for the effect heterogeneity due to a genetic variation from data where the exposure is possibly confounded. The primary aim of the paper is to propose methods for internal replication of the inference so that multiple uncorrelated analyses are considered for the same estimand whose validity depend on different assumptions. The authors call this framework `Triangulation Within a Study'. Although, in the literature the formalization of this framework has been called Evidence Factors analysis; see the book Replication and Evidence Factors in Observational Studies and the references therein.*

- Thank you for alerting us to the Evidence Factor literature. We have included a reference to this work in the discussion and listed a more in-depth investigation of the connection between TWIST and Evidence Factors as further research.

*I agree with much of the presentation and analyses of the paper, but I have several*

*major concerns that I list below.*

Thank you for your positive assessment, and for helping us to tighten up the description of the approach with insightful comments.

***Major Comments:***

*1. Definition of the estimand. I am confused by how the GMTE estimate is*

*defined in section 2, equation (2). I think the definition does not match with*

*what the estimand is supposed to be as explained in the text. What is `g' in equation (2)? This seems like a typo and should be corrected. If the estimand is defined using these marginal expectations in (2), then it would depend on the exposure distribution, i.e., the effect on the popula-*

*tion will be attenuated when a small fraction of the population receives the*

*treatment. Although, as I understand, this is not the aim of this estimand.*

- We agree that the notation used in the Estimand was confusing, although it is similar to the notation used in the mediation analysis literature. See for example equation (1) in Imai, Tingley and Keele (2015) <https://imai.fas.harvard.edu/research/files/BaronKenny.pdf> . In this revised version we have removed any mention of mediation per se and have instead re-defined the causal estimand as a `genetically **moderated** treatment effect’ (GMTE). We now express the GMTE estimand as the average causal effect if everyone could receive moderated treatment level T*=1 (i.e. the full or enhanced effect) versus if everyone could receive treatment level T*=0.

*2. Model (3). You need to make it clear if this parametric model specification is*

*a toy model used for illustration or an assumption for your analyses and results.*

*From the appendices, it seems like this is an assumption for all your technical*

*results. Additionally, when you move from the potential outcomes notation to the ob-*

*served variable Y , please specify the appropriate SUTVA.*

- We now make clear that the estimand and subsequent proofs are valid with respect to the outcome model (2)
- We now go from model (2) to the Estimand in equation (3) rather than vice versa. The estimand is much easier to interpret, since it is the direct coefficient of the variable T* in the model.

*3. After the GMTE estimand is defined using the potential outcomes notation,*

*under the parametric model you need to show that the estimand equates to…In this case, you need to be careful if assumptions are needed to make this*

*connection.*

- *Please see answer to question 2, we hope this is clear now*

*4. Proofs in the Appendices.*

*. In Appendices 1, 2, and 4, `Under model (5) ...' should be `Under model*

*(3) ...'.*

- Thank you, we have corrected this, now the appropriate model is model (2)

*In Appendix 1 (also 6), _GMTE(1)(_) is used without definition.*

- Corrected

*. In Appendix 2, the condition Cov(U;G) | T is trivial and incorrect; Cov(U;G) is just a number. (also in Table 1).*

- Thank you for spotting this. We meant to write that the covariance of U and G given T does not depend on T, or that Cov(U,G)|T=Cov(U,G)

*. In Appendix 1, GMTE(0) is consistent for 0 under PG3 and G ?? U j T = 0. (It should not be in Table 1)*

- Thank you for spotting this typo. Indeed it is consistent for 0 under those stated assumptions. However, since it is an intrinsic component of the RGMTE estimate and it is used within the TWIST framework on its own to assess potential violation of the PG assumptions, we would like to keep it in Table 1. It is now clearly labelled as *not* an estimator of the GMTE.

*5. Homogeneity.*

*I would like a little more clarity regarding how the term `Homogeneity' is used in the paper. Treatment effect homogeneity typically means the treatment effect does not change with an individual. That is, in model (3), treatment effect homogeneity means that beta1=beta0.*

*The paper however uses `Homogeneity' to mean that the treatment effect is only*

*present in the G = 1 group, i.e., beta0=0. Thus, I think clarification is needed.*

- Thank you very much for raising this point. We now clarify the difference between the homogeneity assumption in the general IV setting and in this special case as you suggest (see the newly written section ` The causal estimand and key identifying assumptions’)

*6. Combined estimators { precision vs robustness.}*

*The combination method proposed in Section 2.5 aims to improve the precision of the combined estimate when the individual estimates are deemed similar. This is clearer in the smaller standard errors of the combined estimates relative to the individual estimates in Tables 5 and 6. Given the goal of the framework is to improve the robustness of the inference due to possible violations of variety of assumptions, I wonder if this is the correct goal. If we had a hypothesized value of the estimand, and robustness was the goal, we would not reject it if was not rejected by any of the individual analysis. On the other hand, we would reject a proposed value of the estimand with increased confidence if it is rejected by multiple independent analyses that depend on assumptions that do not completely overlap.*

*Thus, by the duality of CI and testing it seems a robust CI of the estimand by*

*combining multiple estimates should be wider, not narrower. Perhaps a concise discussion is needed to clarify the goal of the combination strategy proposed in the paper.*

- Thank you again for this insightful comment. We agree that gaining precision is just one potential advantage offered by TWIST, but increasing robustness to type I error within a hypothesis testing framework is a separate but equally valid goal. In the discussion we include the development of a rigorous hypothesis testing framework for TWIST and have inserted a comment on this specific point there (giving credit to you for this suggestion).

*Simulation.*

*In table 3 please report for each combination how many times*

*they were deemed to be similar so they can be combined using your assessment by the Q statistic*

- This information has been added to the results in Table 3

*Minor Comments:*

1. *Extra `the' in the first sentence of the second paragraph on page 2.* ***Corrected***
2. *2. In the same paragraph, fill in the missing REF.* ***Corrected***
3. *3. In equation (6), use ^ _GMTE(0) for _GMTE(0).* ***Corrected, equation now in Table 1***

*4. On page 14, `The e4e4 group is now ...' to `The e4e4 group is now ...'. Please*

*be consistent elsewhere, e.g. §4.2* ***Corrected***

*5. In the last but one paragraph of the Discussion, `...both analyses after collating*

*a much larger ...' to `...both analyses after collecting a much larger ...'.* ***Corrected***

*6. It might be good to acknowledge some of the literature on Evidence factors.* ***We have now referenced the recent book on Evidence Factors by Rosenbaum (see earlier comments)***

**Reviewer #2:**

*Summary:*

*The paper reviews the limitations that underlie pharmacogenetic approaches that rely on only treated individuals to estimate “genetically mediated treatment effect” (GMTE). The authors propose that when certain assumptions are violated, the RGMTE estimate that leverages information from untreated individuals can be used to correct for certain biases. The authors propose a framework where uncorrelated estimates, which are derived from methods with different underlying assumptions that are observed to be similar could be combined to improve efficiency. Through a simulation study, the authors provide empirical evidence that verifies the assumptions behind each strategy, and show that their proposed RGMTE estimate is the most robust. Finally, the authors illustrate their approach using two examples: CYP2C19 genetic variants that mediate Clopidogrel efficacy and APOE genetic variants that mediate statin efficacy. This work clearly presents the limitations/assumptions of each GMTE estimation method and provides a practical decision framework for pharmacogenetic researchers to decide on the best strategy. However, further clarifications are needed to address concerns related to simulation results and potential assumption violations in the APOE-statins example. The authors should also expand on examples of prior knowledge that may suggest the violation of assumptions.*

- Thank you for the broadly positive assessment of our manuscript

*Comments:*

1. *Under scenario 2 of the simulation (NUC violation) presented in Table 2, the MR estimate is unbiased but has a very large standard error. Please explain the reason behind this, and clarify whether this has any implications on the use of the MR estimate or the combined RGMTE/MR estimate under such a scenario?*

- Thank you for raising this. The MR estimator is unique among the four estimators because it takes the form of a ratio. It is therefore susceptible to bias and imprecision if the denominator of the ratio estimate is small. Extra text has been added to clarify this point.

2. *Under scenario 4 of the simulation (PG1 and NUC violation) presented in Table 2, the MR estimate is unbiased while the RGMTE estimate is biased. However, Table 3 shows that the combined RGMTE/MR estimate is unbiased (highlighted in bold). Please explain the reason behind this, and clarify whether the approach to combine estimates is still valid under the scenario where one of the estimates is biased due to the violation of assumption(s)?*

- Thank you for spotting this. You are correct that in this scenario that whilst the MR estimate is consistent the RGMTE is not, although its bias is small. For this reason we should not have colour-coded it black in Table 3. This has been corrected.

3. *In section 4.1, CYP2C19 LoF carriers were compared to non-carriers with regards to the risk of ischemic stroke whilst on Clopidogrel. Please clarify if non-carriers include CYP2C19 gain-of-function (e.g. CYP2C19*17) carriers? If so, the estimates would be more interpretable if gain-of-function carriers are excluded.*

- Thank you for raising this. In our analysis we did not stratify the participants by the *17 variant. This decision was based on results from our companion paper (see Supplementary Table 3 of Pilling et al 2021) which showed that `normal’ or wildtype individuals had a near identical risk of stroke than gain-of-function *17 individuals (HR 0.99). For convenience we re-print the table below

| **Outcome / CYP2C19 genotype** | | **N** | **N cases** | **Person-years** | **HR** | **95% CIs** | | ***p*** |
| --- | --- | --- | --- | --- | --- | --- | --- | --- |
| ***Ischemic stroke*** | |  |  |  |  |  |  |  |
|  | Normal (*1/*1) | 2,948 | 37 | 7,208 |  |  |  |  |
|  | Intermediate/poor (any *2-*8) | 1,668 | 33 | 3,919 | 1.61 | 1.01 | 2.58 | 0.047 |
|  | Poor or rapid heterozygote (*2–*8/*17) | 476 | 8 | 1,191 | 1.25 | 0.58 | 2.69 | 0.570 |
|  | Rapid (any *17) | 2,385 | 32 | 6,039 | 0.99 | 0.62 | 1.59 | 0.970 |
|  | *Total* | *7,477* | *110* | *18,358* |  |  |  |  |
| ***Myocardial infarction*** | |  |  |  |  |  |  |  |
|  | Normal (*1/*1) | 2,905 | 707 | 5,403 |  |  |  |  |
|  | Intermediate/poor (any *2-*8) | 1,634 | 442 | 2,805 | 1.16 | 1.03 | 1.30 | 0.017 |
|  | Poor or rapid heterozygote (*2–*8/*17) | 466 | 112 | 904 | 0.98 | 0.81 | 1.20 | 0.880 |
|  | Rapid (any *17) | 2,354 | 562 | 4,691 | 0.95 | 0.85 | 1.06 | 0.330 |
|  | *Total* | *7,359* | *1823* | *13,803* |  |  |  |  |

Removing *17 individuals from the analysis was seen to have minimal impact on point estimation, but a significant loss in precision, because they constitute around 22% of the population. For this reason we have kept the analysis as is, but have now explained this point to the reader. We hope this is acceptable.

4. *Table 6 shows that the frequency of the e2e3 and e3e4 APOE genotype is different in statin users and non-statin users. Please explain if this has any implications on the validity of the different approaches and whether this suggests a violation of the PC1 assumption?*

- Thank you for raising this. Indeed this confirms that genotype is associated with treatment and suggests a violation of assumptions PG1 and or PG2. This is seen in Table 6 and is also highlighted by the GMTE(0) estimate in Table 7. We do not think it is possible to formally test whether PG1 is violated on its own, because this would require testing for a G-T association after conditioning on all measured and unmeasured confounders, unless of course one assumed that NUC was satisfied**.**

5. *In the discussion section, the authors mentioned that “expert knowledge and prior evidence should also be leveraged to decide whether the particular assumptions of the causal estimation strategy are likely to be met”. It would be very helpful to expand on this by providing examples. For example, if a variant is known to be associated with the outcome, then the PG3 assumption required for CAT and MR is likely violated, and the RGMTE estimate should be favored. Also, if it is known that G=0 individuals still benefit from treatment, then the homogeneity assumption is likely violated, and one needs to pay attention to the PG1 assumption when using the MR estimate. Relating to comment 4, could one then check if the PG1 assumption may be violated?*

- Thank you for this excellent suggestion. We have added text to this discussion to include these valuable points.

*Minor Comments*

1. *The simulation results suggest that for RGMTE to be unbiased when either NUC or PG1 are met. For consistency and readability, it would be helpful if this is explicitly mentioned in Table 1 and the top left panel of Figure 2.*

- Thank you, this has been made explicit in Table 1 of the paper in the revised version

2. *There is most likely an error on the bottom right panel of Figure 2. Q > χ2 _GMTE(1),CAT 1,1− α_ . should be replaced with Q < χ2 _GMTE(1),CAT 1,1− α_ .*

- Thanks for spotting this, this has been corrected. This information now forms part of the new Figure 3

3. *There is a typo in Section 4.3 (Page 13): “For brevity, we focus on the results of the e2e2 versus e3e3…” e2e2 should be replaced with e2e3.*

- Corrected, thanks.

**Reviewer #3:**

*The authors aimed to review the methods of causal inference in the pharmacogenetics (PG) studies, especially to evaluate the robustness of the estimates of genetically mediated treatment effect (GMTE) when part or all of the assumptions are violated. They also proposed a new decision framework for the combination of these estimators. The authors showed theoretically and in simulation the robustness of each single and combined estimates under different scenarios. Overall I think the framework is clear. More details could be added to the manuscript to make the logistics clearer. More simulation settings could be added to have a comprehensive evaluation of the methods.*

- Thank you for the positive assessment of our manuscript

*I didn’t fully understand the logic behind using the “Triangulation of evidence” in this decision framework, specifically, is it always the case that the “Triangulation of evidence” is needed in this framework? Can the authors give a more detailed explanation in the introduction?*

- Thank you for raising this. We have added further text to the end of the introduction to clarify how Triangulation would work in practice.

*Page 3 line 3 “across across” → “across”*

- Corrected

*Page 3 paragraph 2 line 2 “the its effect” → “the effect”*

- Corrected

*Page 3 paragraph 2 line 5 what does the REF in the parenthesis mean*

- Typo removed

*Page 4 paragraph 1, in the definition of Ti(j), does the author mean that the treatment assignment depends on the genotype j? Isn’t this a violation of the PG assumption?*

- We are sorry for the confusion. No we did not mean that treatment assignment T depends on genotype G (which would be a violation of PG1 as you point out but rather that the treatment level experienced depends on T and G.

*Page 4 paragraph 4, “in equation (5) is zero” → “equation (4)”*

- Corrected

*Caption of figure 2, the “CAT estimates” where CAT is not explicitly stated anywhere before.*

- Thank you for pointing this out, we have spelled out the acronym to avoid confusion. Figure 2 has now been changed in the paper as we thought it did not add to the clarity of the message

*Page 5 Equation (6), can you make the order of G and T the same in both terms?*

- Thanks for spotting, this has been corrected, but the equation has also been removed from the main body of the paper.

*In the simulation study, how would the lower frequencies (<0.5) of the genotype influence the performances of the estimators?*

- Thank you for raising this point. The frequency of the genotype will indeed effect the efficiency of each estimator in different ways, but as a general rule all methods will be less efficient as the allele frequency becomes rarer. We have included an additional simulation study to assess how the precision of each estimator is affected by decreasing allele frequency (see new Figure 5). The results show that the CAT and MR estimators are most adversely affected, precisely because they are ratio estimates, where the denominator depends on G. Note that we erroneously stated that the allele frequency for the main simulation study was set to 0.5. It was in fact set to 0.3. This has been corrected.

*Section 3, how were the Z simulated? Would Z affect the T?*

- Thank you for spotting this oversight, Z was simulated as a standard normal covariate, which is now shown in the data generating model of Section 3. Yes, Z affects both T and Y but is not directly affected by G in the model.

*Figure 4, any difference between the solid and dotted lines?*

- In a previous version of the simulation study the green line showing the distribution of MR estimates overlapped to the extent that a dotted line was needed to distinguish it. It has now been made solid as it is clearly unnecessary here.

*Figure 5 and 6, what are the red dots?*

- The red dots show the GMTE(0) estimate. Since this is an estimate for the bias of the GMTE(1) estimate and not a GMTE estimate itself, we thought it appropriate to denote it with a different colour. This has now been made clear in the figure legends

*Reviewer #4: The triangulation aspect of this paper is an excellent contribution to the literature. Triangulation within studies avoids many of the problems with triangulation between studies, most important, heterogeneity between populations. The careful attention to the assumptions required for each estimator and how violations bias which estimators is precisely how we need to think about triangulation going forward.*

- Thank you for your positive assessment of our work

*I have some comments on many aspects of the paper which I hope will contribute to its quality.

Figure 1
-I realize the box says typical but NUC is not sufficient. Exchangeability (which includes NUC and selection bias and other potential biases) is sufficient. I know there is less general familiarity with that term.*

- Thank you for pointing this out. We have added a sentence and an additional reference in the introduction to make this clear, and have also modified the legend to Figure 1 to stress this point again.

*-The IV assumptions are also not technically complete. It is not enough that the IV be independent of X-Y confounders, it must be exchangeable with Y. The latter term also considers possible G-Y confounders that are not related to X.*

- The third IV assumption we invoke (IV3) is that G independent of Y given X and U. Would this not rule out the existence of G-Y confounders not related to X? We have modified text at the start of the introduction to try to encompass this point, however.

*-T* is not defined until later in the text making it confusing for the reader*

- We have re-written the text considerably in our revised version and are now much more explicit about the definition of T* (see new Section 2.1)

*About the definition of the target parameter:*

*-This is the clearest definition of the estimand, to me: “The GMTE is equal to the difference in treatment effects experienced by the two genetic groups, β_1- β_0.” But I would write this estimand as: (E[Y_i (G=1,T=1)]-E[Y_i (G=1,T=0)])-(E[Y_i (G=0,T=1)]-E[Y_i (G=0,T=0)])*

- Thank you for raising this point. Other reviewers also queried the form of our original estimand. In our revised version we have re-written the estimand so that it is a direct function of T*. We feel this definition is clearer and flows directly from model (2) in Section 2.1.

*-Equation 2, to me, is not the correct estimand. It is a type of indirect effect. It compares 1) the value of Y if G takes value g and T takes the value it would have taken if G was set to 1, to 2) the value of Y if G takes value g and T takes the value it would have taken if G was set to 0. If G does not cause T, T(1)=T(0) which means the entire equation equals 0.*

- Please see the new form of the estimand and our previous answer.

*-the term "genetically mediated" suggests that G is the mediator here which it is not. It is interacting with T, not mediating its effect. I would suggest an alternative name.*

- Thank you. We now call the estimand the `Genetically Moderated Treatment Effect’

*Other comments:
-It might make the explanation clearer to the reader to point out that Equation 4 is just a straightforward causal model with an interaction.*

- This has been added to Section 2.1

*-If the treatment effect is 0 among G=0, how does this imply that the homogeneity assumption is satisfied? The causal effect of T among those with G=0 is 0 and if the causal effect of T in G=1 is non-zero, this implies that homogeneity is not satisfied.*

- Please see revised Section 2.1. Following comments from another reviewer we have tightened up the language in relation to the homogeneity assumption, distinguishing its meaning within a standard IV/MR analysis and our use of it within the TWIST framework.

*In addition, because of the way these data are generated, even a violation of homogeneity should not bias the MR estimate (because the relationship between the IV and the exposure is not also modified).*

- We agree. In Table 1 we state that either the Homogeneity assumption or the PG1 assumption are required, along with other combinations of assumptions in order for the MR estimate to be consistent, but not both.

*-The term causal DAG is used for the first time on page 4 but is never defined. Many of the diagrams in Figure in 1 are not true causal DAGs because the edges are not all directed (they have no arrow head).*

- We have removed the term `DAG’ from the paper and replaced with `causal diagram’. We have also modified the causal diagram figure 2 so that there are now no undirected edges.

*-Correct me if I’m wrong, but the “robust” estimator only works if there is no effect modification by other variables associated with T. If there are, then b_PG2 and b_PG3 will not be the same across strata of T.*

- Thank you, yes we believe this is correct. One way in which effect modification could be induced is when PG1 and NUC are violated, because conditioning on treatment T then moderates the strength of association between G and U and consequently G and Y. We label this bias b_{PG1} in Section 2.4. We have amended our description of the robust estimator and borrowed your suggested language to make this clear.

*-Section 2.2. I’m confused because it seems that T* is defined as the interaction of T and G so I would assume it is only 1 when T=1 and G=1. But then it is described as the de-facto exposure which would lead me to believe that it is just T.*

- We are sorry for the confusion, you are correct that T*=1 iff G=1 and T=1. We have modified the description of the model and estimand so that this is hopefully now clear.

*-It would be nice if there was more discussion of the consequences of using significance tests to make these decisions. Personally, I prefer making these judgements based on subject matter knowledge rather than significance tests (the authors do acknowledge the importance of subject matter knowledge in all of this). I’m worried, particularly when there is an easy-to-use R package, that some researchers will simply plug in their data and report whatever result comes out the other end without considering the shortcomings of significance tests in the context of heterogeneity tests and this type of decision making.*

- Thank you for raising this point which was also picked up on by another reviewer. In the revised version we have tried to stress the use of the TWIST framework for quantitatively and qualitatively combining results from different estimation strategies that make non-overlapping sets of assumptions. Although we present a possible framework for the quantitatively combining two or more estimators, we now stress the importance of weighing up the plausibility of each assumption too. This may rule out one or more estimators altogether

*-On that note, is it not worth mentioning the low power of heterogeneity tests? Particularly when sequential heterogeneity tests are performed this can easily lead to an incorrect decision, no?*

- Following a request from another reviewer we now report the statistical power to reject the heterogeneity test statistics for the combined estimators across simulation scenarios 1-6 in Table 3. In the discussion we list the development of a full hierarchical testing procedure for TWIST as further work. Ensuring that such a testing procedure maintains FWER or FDR control will inevitably lead to a loss of power.

*One important aspect that I feel is underdiscussed in this manuscript is the correlation between systematic errors in these estimators. The authors have reported which estimators are statistically uncorrelated which is a measure of correlation between random error and have relied on homogeneity tests to decide whether estimates agree with the assumption being that when they agree they’re unbiased. But it’s possible that systematic biases are correlated which can lead to rough agreement between estimates even when they’re both biased. This is why the (small) triangulation literature talks about orthogonal biases. For example, when GMTE(1) and MR are biased they are biased downwards meaning that they have a bias that is correlated. In the simulation this did not lead to pooling of the estimates when they should not have been pooled. But one can imagine scenarios with smaller sample sizes and different sets of bias parameters where estimates roughly agree (and pass the homogeneity test) but are both biased.*

- Thank you for alerting us to this important point. We completely agree. Although we saw no evidence of this in simulation scenarios 4-6, this could be an issue in other contexts and it is too simplistic to equate similarity of two estimates as confirmation they are correct. We have added a paragraph to the discussion section to make this point and have listed an investigation of this topic as further work.

*Small comments:*

*Reference missing in second paragraph of page 3* **Corrected**
*Appendix 3, equation 13: the denominator seems incorrect* **Corrected**
