# Supplementary material for: The Triangulation WIthin a STudy (TWIST) framework for causal inference within pharmacogenetic research
Source: PLoS Genet. 2021 Sep 8;17(9):e1009783. doi: 10.1371/journal.pgen.1009783 (PMC8452063; doi:10.1371/journal.pgen.1009783)
Supplement: Peer review history

# Peer review: Decision Letter 0

**DOI:** 10.1371/journal.pgen.1009783.r001

**Authors:** David Balding, Zoltán Kutalik

23 Jun 2021

Dear Dr Bowden,

Thank you very much for submitting your Methods entitled 'The Triangulation WIthin A STudy (TWIST) framework for causal inference within Pharmacogenetic research' to PLOS Genetics.

The manuscript was fully evaluated at the editorial level and by independent peer reviewers. The reviewers appreciated the attention to an important problem, but raised some substantial concerns about the current manuscript. Based on the reviews, we will not be able to accept this version of the manuscript, but we would be willing to review a much-revised version. We cannot, of course, promise publication at that time.

Should you decide to revise the manuscript for further consideration here, your revisions should address the specific points made by each reviewer. We will also require a detailed list of your responses to the review comments and a description of the changes you have made in the manuscript.

If you decide to revise the manuscript for further consideration at PLOS Genetics, please aim to resubmit within the next 60 days, unless it will take extra time to address the concerns of the reviewers, in which case we would appreciate an expected resubmission date by email to plosgenetics@plos.org.

If present, accompanying reviewer attachments are included with this email; please notify the journal office if any appear to be missing. They will also be available for download from the link below. You can use this link to log into the system when you are ready to submit a revised version, having first consulted our Submission Checklist.

To enhance the reproducibility of your results, we recommend that you deposit your laboratory protocols in protocols.io, where a protocol can be assigned its own identifier (DOI) such that it can be cited independently in the future. Additionally, PLOS ONE offers an option to publish peer-reviewed clinical study protocols. Read more information on sharing protocols at https://plos.org/protocols?utm_medium=editorial-email&utm_source=authorletters&utm_campaign=protocols

Please be aware that our data availability policy requires that all numerical data underlying graphs or summary statistics are included with the submission, and you will need to provide this upon resubmission if not already present. In addition, we do not permit the inclusion of phrases such as "data not shown" or "unpublished results" in manuscripts. All points should be backed up by data provided with the submission.

While revising your submission, please upload your figure files to the Preflight Analysis and Conversion Engine (PACE) digital diagnostic tool.  PACE helps ensure that figures meet PLOS requirements. To use PACE, you must first register as a user. Then, login and navigate to the UPLOAD tab, where you will find detailed instructions on how to use the tool. If you encounter any issues or have any questions when using PACE, please email us at figures@plos.org.

PLOS has incorporated Similarity Check, powered by iThenticate, into its journal-wide submission system in order to screen submitted content for originality before publication. Each PLOS journal undertakes screening on a proportion of submitted articles. You will be contacted if needed following the screening process.

To resubmit, use the link below and 'Revise Submission' in the 'Submissions Needing Revision' folder.

[LINK]

We are sorry that we cannot be more positive about your manuscript at this stage. Please do not hesitate to contact us if you have any concerns or questions.

Yours sincerely,

Zoltán Kutalik, PhD

Associate Editor

PLOS Genetics

David Balding

Section Editor: Methods

PLOS Genetics

Editor comments:

The reviewers agree that within-study triangulation is an important part of the literature and the authors' contribution is useful. The presented decision framework is novel and a key first step, which should be further developed in the future (to be able to associate rigorous type I error rate to decisions based on such trees). Reviewers also appreciated the relevance of the application.

However, all reviewers raised important points to be addressed and several key clarifications to be able to better judge the advances this work represents. Since the target audience of the paper may be quite narrow, in order to reach out to the wide readership of PLoS Genetics, the languages could be made more accessible.

Reviewer's Responses to Questions

**Comments to the Authors:**

Reviewer #1: Review is uploaded as an attachment.

Reviewer #2: The review is uploaded as an attachment.

Reviewer #3: The authors aimed to review the methods of causal inference in the pharmacogenetics (PG) studies, especially to evaluate the robustness of the estimates of genetically mediated treatment effect (GMTE) when part or all of the assumptions are violated. They also proposed a new decision framework for the combination of these estimators. The authors showed theoretically and in simulation the robustness of each single and combined estimates under different scenarios. Overall I think the framework is clear. More details could be added to the manuscript to make the logistics clearer. More simulation settings could be added to have a comprehensive evaluation of the methods. My comments are as follows.

I didn’t fully understand the logic behind using the “Triangulation of evidence” in this decision framework, specifically, is it always the case that the “Triangulation of evidence” is needed in this framework? Can the authors give a more detailed explanation in the introduction?

Page 3 line 3 “across across” → “across”

Page 3 paragraph 2 line 2 “the its effect” → “the effect”

Page 3 paragraph 2 line 5 what does the REF in the parenthesis mean

Page 4 paragraph 1, in the definition of Ti(j), does the author mean that the treatment assignment depends on the genotype j? Isn’t this a violation of the PG assumption?

Page 4 paragraph 4, “in equation (5) is zero” → “equation (4)”

Caption of figure 2, the “CAT estimates” where CAT is not explicitly stated anywhere before.

Page 5 Equation (6), can you make the order of G and T the same in both terms?

In the simulation study, how would the lower frequencies (<0.5) of the genotype influence the performances of the estimators?

Section 3, how were the Z simulated? Would Z affect the T?

Figure 4, any difference between the solid and dotted lines?

Figure 5 and 6, what are the red dots?

Reviewer #4: The triangulation aspect of this paper is an excellent contribution to the literature. Triangulation within studies avoids many of the problems with triangulation between studies, most important, heterogeneity between populations. The careful attention to the assumptions required for each estimator and how violations bias which estimators is precisely how we need to think about triangulation going forward.

I have some comments on many aspects of the paper which I hope will contribute to its quality.

Figure 1

-I realize the box says typical but NUC is not sufficient. Exchangeability (which includes NUC and selection bias and other potential biases) is sufficient. I know there is less general familiarity with that term.

-The IV assumptions are also not technically complete. It is not enough that the IV be independent of X-Y confounders, it must be exchangeable with Y. The latter term also considers possible G-Y confounders that are not related to X

-T* is not defined until later in the text making it confusing for the reader

About the definition of the target parameter:

-This is the clearest definition of the estimand, to me: “The GMTE is equal to the difference in treatment effects experienced by the two genetic groups, β_1- β_0.” But I would write this estimand as: (E[Y_i (G=1,T=1)]-E[Y_i (G=1,T=0)])-(E[Y_i (G=0,T=1)]-E[Y_i (G=0,T=0)])

-Equation 2, to me, is not the correct estimand. It is a type of indirect effect. It compares 1) the value of Y if G takes value g and T takes the value it would have taken if G was set to 1, to 2) the value of Y if G takes value g and T takes the value it would have taken if G was set to 0. If G does not cause T, T(1)=T(0) which means the entire equation equals 0.

-the term "genetically mediated" suggests that G is the mediator here which it is not. It is interacting with T, not mediating its effect. I would suggest an alternative name.

Other comments:

-It might make the explanation clearer to the reader to point out that Equation 4 is just a straightforward causal model with an interaction.

-If the treatment effect is 0 among G=0, how does this imply that the homogeneity assumption is satisfied? The causal effect of T among those with G=0 is 0 and if the causal effect of T in G=1 is non-zero, this implies that homogeneity is not satisfied. In addition, because of the way these data are generated, even a violation of homogeneity should not bias the MR estimate (because the relationship between the IV and the exposure is not also modified).

-The term causal DAG is used for the first time on page 4 but is never defined. Many of the diagrams in Figure in 1 are not true causal DAGs because the edges are not all directed (they have no arrow head).

-Correct me if I’m wrong, but the “robust” estimator only works if there is no effect modification by other variables associated with T. If there are, then b_PG2 and b_PG3 will not be the same across strata of T.

-Section 2.2. I’m confused because it seems that T* is defined as the interaction of T and G so I would assume it is only 1 when T=1 and G=1. But then it is described as the de-facto exposure which would lead me to believe that it is just T.

-It would be nice if there was more discussion of the consequences of using significance tests to make these decisions. Personally, I prefer making these judgements based on subject matter knowledge rather than significance tests (the authors do acknowledge the importance of subject matter knowledge in all of this). I’m worried, particularly when there is an easy-to-use R package, that some researchers will simply plug in their data and report whatever result comes out the other end without considering the shortcomings of significance tests in the context of heterogeneity tests and this type of decision making.

-On that note, is it not worth mentioning the low power of heterogeneity tests? Particularly when sequential heterogeneity tests are performed this can easily lead to an incorrect decision, no?

One important aspect that I feel is underdiscussed in this manuscript is the correlation between systematic errors in these estimators. The authors have reported which estimators are statistically uncorrelated which is a measure of correlation between random error and have relied on homogeneity tests to decide whether estimates agree with the assumption being that when they agree they’re unbiased. But it’s possible that systematic biases are correlated which can lead to rough agreement between estimates even when they’re both biased. This is why the (small) triangulation literature talks about orthogonal biases. For example, when GMTE(1) and MR are biased they are biased downwards meaning that they have a bias that is correlated. In the simulation this did not lead to pooling of the estimates when they should not have been pooled. But one can imagine scenarios with smaller sample sizes and different sets of bias parameters where estimates roughly agree (and pass the homogeneity test) but are both biased.

Small comments:

Reference missing in second paragraph of page 3

Appendix 3, equation 13: the denominator seems incorrect

**********

**Have all data underlying the figures and results presented in the manuscript been provided?**

Large-scale datasets should be made available via a public repository as described in the *PLOS Genetics* data availability policy, and numerical data that underlies graphs or summary statistics should be provided in spreadsheet form as supporting information.

Reviewer #1: Yes

Reviewer #2: None

Reviewer #3: **No: **In simulation study, the data generation part is incomplete. The UKBB data is restricted by license.

Reviewer #4: Yes

**********

PLOS authors have the option to publish the peer review history of their article (what does this mean?). If published, this will include your full peer review and any attached files.

If you choose “no”, your identity will remain anonymous but your review may still be made public.

**Do you want your identity to be public for this peer review?** For information about this choice, including consent withdrawal, please see our Privacy Policy.

Reviewer #1: No

Reviewer #2: No

Reviewer #3: No

Reviewer #4: **Yes: **Jeremy Labrecque

Attachment Submitted filename: PGENETICS-D-21-00581_reviewer.docx Click here for additional data file.

Attachment Submitted filename: review_PGENETICS-D-21-00581.pdf Click here for additional data file.

# Author comment: Author response to Decision Letter 0

**DOI:** 10.1371/journal.pgen.1009783.r002

22 Jul 2021

Attachment Submitted filename: Response to reviewers.docx Click here for additional data file.

# Peer review: Decision Letter 1

**DOI:** 10.1371/journal.pgen.1009783.r003

**Authors:** David Balding, Zoltán Kutalik

17 Aug 2021

Dear Dr Bowden,

We are pleased to inform you that your manuscript entitled "The Triangulation WIthin A STudy (TWIST) framework for causal inference within Pharmacogenetic research" has been editorially accepted for publication in PLOS Genetics. Congratulations!

Before your submission can be formally accepted and sent to production you will need to complete our formatting changes, which you will receive in a follow up email. Please be aware that it may take several days for you to receive this email; during this time no action is required by you. Please note: the accept date on your published article will reflect the date of this provisional acceptance, but your manuscript will not be scheduled for publication until the required changes have been made.

Once your paper is formally accepted, an uncorrected proof of your manuscript will be published online ahead of the final version, unless you’ve already opted out via the online submission form. If, for any reason, you do not want an earlier version of your manuscript published online or are unsure if you have already indicated as such, please let the journal staff know immediately at plosgenetics@plos.org.

In the meantime, please log into Editorial Manager at https://www.editorialmanager.com/pgenetics/, click the "Update My Information" link at the top of the page, and update your user information to ensure an efficient production and billing process. Note that PLOS requires an ORCID iD for all corresponding authors. Therefore, please ensure that you have an ORCID iD and that it is validated in Editorial Manager. To do this, go to ‘Update my Information’ (in the upper left-hand corner of the main menu), and click on the Fetch/Validate link next to the ORCID field.  This will take you to the ORCID site and allow you to create a new iD or authenticate a pre-existing iD in Editorial Manager.

If you have a press-related query, or would like to know about making your underlying data available (as you will be aware, this is required for publication), please see the end of this email. If your institution or institutions have a press office, please notify them about your upcoming article at this point, to enable them to help maximise its impact. Inform journal staff as soon as possible if you are preparing a press release for your article and need a publication date.

Thank you again for supporting open-access publishing; we are looking forward to publishing your work in PLOS Genetics!

Yours sincerely,

Zoltán Kutalik, PhD

Associate Editor

PLOS Genetics

David Balding

Section Editor: Methods

PLOS Genetics

www.plosgenetics.org

Twitter: @PLOSGenetics

----------------------------------------------------

Comments from the Editors

Reviewers 2 and 3 have raised minor points, which we ask you to address as you see fit in preparing the final version for publication, there is no requirement for any further editorial review.

Comments from the Reviewers

Reviewer #1: The authors have addressed all comments and issues raised. They have made relevant changes to the manuscript. I have no further comments.

Reviewer #2: I have provided my comments in the attached document.

Reviewer #3: I only have one minor comment:

Throughout the method session, I feel like you are using U to represent all confounders (both observed and unobserved), which is a little confusing because you have previously denoted unobserved confounders as U. I would suggest you use different notation for observed and unobserved confounders, and a third notation for both of them.

Reviewer #4: Thank you to the authors for carefully considering and implementing my previous suggestions. I have no further comments.

**********

**Have all data underlying the figures and results presented in the manuscript been provided?**

Large-scale datasets should be made available via a public repository as described in the *PLOS Genetics* data availability policy, and numerical data that underlies graphs or summary statistics should be provided in spreadsheet form as supporting information.

Reviewer #1: None

Reviewer #2: None

Reviewer #3: Yes

Reviewer #4: Yes

**********

PLOS authors have the option to publish the peer review history of their article (what does this mean?). If published, this will include your full peer review and any attached files.

If you choose “no”, your identity will remain anonymous but your review may still be made public.

**Do you want your identity to be public for this peer review?** For information about this choice, including consent withdrawal, please see our Privacy Policy.

Reviewer #1: No

Reviewer #2: No

Reviewer #3: No

Reviewer #4: **Yes: **Jeremy Labrecque

----------------------------------------------------

**Data Deposition**

If you have submitted a Research Article or Front Matter that has associated data that are not suitable for deposition in a subject-specific public repository (such as GenBank or ArrayExpress), one way to make that data available is to deposit it in the Dryad Digital Repository. As you may recall, we ask all authors to agree to make data available; this is one way to achieve that. A full list of recommended repositories can be found on our website.

The following link will take you to the Dryad record for your article, so you won't have to re‐enter its bibliographic information, and can upload your files directly: 

http://datadryad.org/submit?journalID=pgenetics&manu=PGENETICS-D-21-00581R1

More information about depositing data in Dryad is available at http://www.datadryad.org/depositing. If you experience any difficulties in submitting your data, please contact help@datadryad.org for support.

Additionally, please be aware that our data availability policy requires that all numerical data underlying display items are included with the submission, and you will need to provide this before we can formally accept your manuscript, if not already present.

----------------------------------------------------

**Press Queries**

If you or your institution will be preparing press materials for this manuscript, or if you need to know your paper's publication date for media purposes, please inform the journal staff as soon as possible so that your submission can be scheduled accordingly. Your manuscript will remain under a strict press embargo until the publication date and time. This means an early version of your manuscript will not be published ahead of your final version. PLOS Genetics may also choose to issue a press release for your article. If there's anything the journal should know or you'd like more information, please get in touch via plosgenetics@plos.org.

Attachment Submitted filename: review_PGENETICS-D-21-00581_R1_reviewer.pdf Click here for additional data file.

# Editor report: Acceptance letter

**DOI:** 10.1371/journal.pgen.1009783.r004

**Authors:** David Balding, Zoltán Kutalik

3 Sep 2021

PGENETICS-D-21-00581R1 

The Triangulation WIthin A STudy (TWIST) framework for causal inference within Pharmacogenetic research 

Dear Dr Bowden, 

We are pleased to inform you that your manuscript entitled "The Triangulation WIthin A STudy (TWIST) framework for causal inference within Pharmacogenetic research" has been formally accepted for publication in PLOS Genetics! Your manuscript is now with our production department and you will be notified of the publication date in due course.

The corresponding author will soon be receiving a typeset proof for review, to ensure errors have not been introduced during production. Please review the PDF proof of your manuscript carefully, as this is the last chance to correct any errors. Please note that major changes, or those which affect the scientific understanding of the work, will likely cause delays to the publication date of your manuscript. 

Soon after your final files are uploaded, unless you have opted out or your manuscript is a front-matter piece, the early version of your manuscript will be published online. The date of the early version will be your article's publication date. The final article will be published to the same URL, and all versions of the paper will be accessible to readers.

Thank you again for supporting PLOS Genetics and open-access publishing. We are looking forward to publishing your work! 

With kind regards,

Amy Kiss

PLOS Genetics

On behalf of:

The PLOS Genetics Team

Carlyle House, Carlyle Road, Cambridge CB4 3DN | United Kingdom

plosgenetics@plos.org | +44 (0) 1223-442823

plosgenetics.org | Twitter: @PLOSGenetics
